# A complete chromosome substitution mapping panel reveals genome-wide epistasis in Arabidopsis

**DOI:** 10.1038/s41437-024-00705-1

**Published:** 2024-07-09

**Authors:** Cris L. Wijnen, Ramon Botet, José van de Belt, Laurens Deurhof, Hans de Jong, C. Bastiaan de Snoo, Rob Dirks, Martin P. Boer, Fred A. van Eeuwijk, Erik Wijnker, Joost J. B. Keurentjes

**Affiliations:** 1 Wageningen University and Research, Laboratory of Genetics, Wageningen, The Netherlands; 2 Rijk Zwaan, Molecular Biology Research, Fijnaart, The Netherlands; 3 Managerial Genetics Consulting, Maaseik, Belgium; 4 Wageningen University and Research, Biometris, Wageningen, The Netherlands

**Keywords:** Epistasis, Plant genetics, Genetic linkage study, Quantitative trait loci

## Abstract

Chromosome substitution lines (CSLs) are tentatively supreme resources to investigate non-allelic genetic interactions. However, the difficulty of generating such lines in most species largely yielded imperfect CSL panels, prohibiting a systematic dissection of epistasis. Here, we present the development and use of a unique and complete panel of CSLs in *Arabidopsis thaliana*, allowing the full factorial analysis of epistatic interactions. A first comparison of reciprocal single chromosome substitutions revealed a dependency of QTL detection on different genetic backgrounds. The subsequent analysis of the complete panel of CSLs enabled the mapping of the genetic interactors and identified multiple two- and three-way interactions for different traits. Some of the detected epistatic effects were as large as any observed main effect, illustrating the impact of epistasis on quantitative trait variation. We, therefore, have demonstrated the high power of detection and mapping of genome-wide epistasis, confirming the assumed supremacy of comprehensive CSL sets.

## One sentence summary

Development of a complete panel of chromosome substitution lines enables high power mapping of epistatic interactions in *Arabidopsis thaliana*.

## Introduction

The identification of genetic factors involved in the regulation of quantitative traits is conventionally performed by linkage analysis of genotype-phenotype relationships in segregating mapping populations (Bazakos et al. 2017; Wijnen and Keurentjes 2014). Traditional mapping populations are typically the result of random recombination and segregation of two genotypes in the offspring of an intraspecific cross. Such an approach, however, suffers from a number of inherent complicating factors. These include, amongst others, the simultaneous segregation of multiple quantitative trait loci (QTL) and genetic interactions between them, features that are characteristic for complex polygenic traits. As a result, conventional mapping populations, such as recombinant inbred lines (RILs), require a large collection of segregating lines to obtain sufficient statistical power to unequivocally detect QTLs and epistasis (Bazakos et al. 2017; Bergelson and Roux 2010). Alternatively, chromosome substitution lines may offer a powerful mapping resource for the systematic dissection of epistatic interactions (Singer et al. 2004; Spiezio et al. 2012).

Chromosome substitution lines (CSLs), a.k.a. consomic strains in non-plant species, differ from established mapping populations by their lack of intra-chromosomal recombination. Consequently, CSLs consist of an assembly of non-recombinant chromosomes, each derived from either one of two genetically different parents (Nadeau et al. 2000; Singer et al. 2004). Genetic variation in CSL populations thus depends exclusively on the reshuffling of complete genotypically distinct chromosomes. As a consequence, the maximum size of chromosome substitution panels, i.e. all possible combinations of chromosomes, is finite (2^n^, where n is the haploid chromosome number), depending solely on the chromosome number of the subjected species. Complete sets of CSLs offer the advantage of fully balanced allele frequency distributions, providing equal haplotype class sizes in epistatic analyses, and a relatively small population size for species with low chromosome numbers, allowing high line replication in experiments. To date, a nearly complete set of CSLs has only been established in *Drosophila melanogaster*, due to the ease of generating CSLs and the limited chromosome number in this species (Seiger 1966). However, for most other species, complete sets of CSLs are notoriously difficult to generate using conventional backcross approaches and, despite their promises, only a very limited number of CSLs in just a handful of vertebrate and plant species have been developed (Cowley et al. 2004; Koumproglou et al. 2002; Kuspira and Unrau 1957; Nadeau et al. 2000; Seiger 1966). Moreover, all these existing panels consist of CSLs with an introgression of only a single donor chromosome in a recurrent genetic background, which considerably restricts the analysis of epistatic interactions. Nonetheless, single-chromosome substitution lines (sCSLs) allow the straightforward detection of additive main effects of introgressed chromosomes, while a deviation of the cumulative sum of these effects from the wild type donor phenotype might indicate the presence of epistatic interactions (Spiezio et al. 2012). However, the exact strength and genetic architecture of epistasis can only be decomposed by investigating the combined effect of multiple chromosome substitutions.

**Fig. 1:**
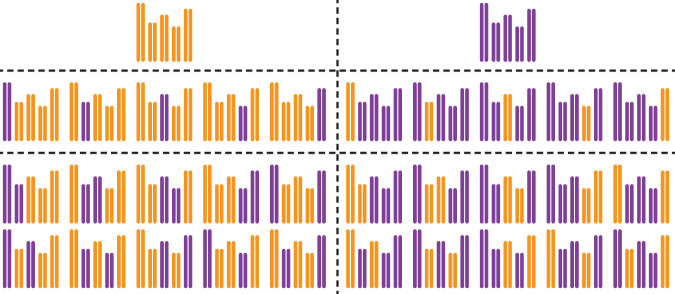
A complete set of *Arabidopsisthaliana* chromosome substitution lines. The complete panel of 32 CSLs can be divided into two reciprocal recurrent backgrounds (vertical dashed line) and subgroups of parental genotypes, single CSLs and CSLs in which two chromosomes are exchanged (horizontal dashed lines). Arabidopsis genomes of each of the CSLs are represented by five homozygous chromosomes derived from either the Col-0 (orange) or L*er* (purple) accession.

The recently emerged reverse breeding technology in Arabidopsis determined a major step forward for the development of CSLs (Wijnen and Keurentjes 2014; Wijnker et al. 2012). This approach makes use of the random segregation of non-recombinant chromosomes to the gametes of achiasmatic hybrids, resulting from the transgenic repression of recombination. These gametes are then converted into haploid offspring through crossing to a haploid inducer line (Ravi and Chan 2010). Finally, the haploid progeny, which consist of an assembly of non-recombinant chromosomes, each derived from either one of the two parents of the initial hybrid, is converted into immortal doubled haploids (DHs). DH seeds occur spontaneously in haploid plants at a low frequency either due to the merging of incidental unreduced gametes that arise by chance, or by somatic doubling. The CSLs produced in this way are now normal diploids containing completely homozygous pairs of chromosomes descending from either parent but in a cytoplasmic background of the haploid inducer line. This approach allows for the generation of complete CSL panels in only three generations, compared to eight to ten generations of inbreeding for RILs. Statistically, the chance of generating any specific CSL is 1/2^n^, which means that only a limited number of DH lines (~3 × 2^n^) need to be genotyped with a relatively low number of markers (typically 3n) to select all possible CSLs (Wijnker et al. 2014). In Arabidopsis, encompassing five chromosome pairs, a complete biparental panel of all possible CSLs comprises 32 (=2^5^) different genotypes (Fig. 1).

## Materials and methods

### Development of chromosome substitution lines

Chromosome substitution lines were obtained from crosses between inbred parental lines as previously described (Wijnker et al. 2012). In brief, semi-sterile Col-0 *RNAi:DMC1* transformed plants, that are impaired in crossover formation, were crossed with wild-type L*er* (CS20) plants to produce achiasmatic F_1_ offspring. F_1_ plants were then crossed to *GFP-TAILSWAP*, a haploid inducer line, to generate F_1_-derived haploids and subsequently doubled haploids (Ravi and Chan 2010). A number of genotypes that were not obtained by the described approach were acquired by specific crosses between generated CSLs or between CSLs and parental lines, whether or not containing the *RNAi:DMC1* construct.

### Confirmation of genotypes

Potential CSLs were genotyped with a set of 151 SNP markers using KASPar assays (Supplementary Tables S3 and S4). These markers covered about 120 Mbp of the total Arabidopsis genome. 95% of the marker intervals were smaller than 2.5 Mbp, which should be sufficient to detect incidental recombinant progeny. To exclude possible phenotypic effects of the *RNAi:DMC1* construct, the absence of the construct in the final selected CSLs was confirmed by additional PCR markers (Wijnker et al. 2014). During propagation we noted two CSLs (Chr1^L*er*^/Chr2^Col^/Chr3^L*er*^/Chr4^Col^/Chr5^Col^ and Chr1^L*er*^/Chr2^L*er*^/Chr3^L*er*^/Chr4^Col^/Chr5^Col^) exhibiting high intra-line variation. Flow cytometry indicated occasional aneuploidy, suggesting that the plants still carried the RNAi-transgene. Data of these genotypes were excluded from further analyses and the CSLs in the panel were replaced by non-transgenic lines. Removal of these two lines during the data analyses caused non-significant allele frequency distortions of 3.3% at max. For all 32 genotypes of the complete CSL panel, construct-free CSLs are now available.

### Development of near isogenic lines

Near isogenic lines were acquired by backcrossing the sCSLs to the recurrent parent and by crossing the resulting F_1_ to the haploid inducer *GFP-TAILSWAP*. Since the F_1_ were transgene-free, it allowed to obtain doubled haploid lines that recombined for a single chromosome. Subsequent genotyping was performed with part of the markers that were available for the confirmation of the CSL genotypes (Supplementary Tables S3 and S4). This allowed to fine-map regions with an approximate resolution of 5 cM (~2.5 Mbp).

### Propagation

To avoid batch differences introduced by generating CSLs in different series of experiments all lines were first propagated simultaneously in a climate chamber. Seeds were sown on wet filter paper and placed at 4 °C in the dark for four days to break residual dormancy and ensure uniform germination. After four days in the cold, plates were transferred to a climate cell at 20 °C in the light. After two days, at radicle protrusion, germinating seeds were transferred to 4 × 4 cm Rockwool blocks in a climate cell set at long day conditions (16 h/8 h, 20/18 °C, day/night). Relative humidity was set to 70% and watering was performed automatically with a Hyponex nutrient solution using a flooding system that bottom watered the Rockwool blocks. Five replicates per genotype were sown, and after germination, these were reduced to three well-established replicates. After two weeks of growth, single-leaf samples were taken for genotyping using three KASP-assays per chromosome (Supplementary Table S4). In addition, a PCR for detecting the presence of the *RNAi:DMC1* construct was performed (Wijnker et al. 2014). Mature plants were dried and only a single plant was harvested per genotype, which served as the seed stock for the following mapping experiment or any further future experimentation.

### Phenotyping experiment

The complete CSL panel was grown in twelve replicates in parallel with three replicates of 100 RILs (Supplementary Tables S5 and S6), obtained from the ABRC stock centre (https://abrc.osu.edu/). The handling of the seeds and growth conditions were similar to the propagation conditions, with the exception of short day growing conditions (8 h/16 h, 20/18 °C, day/night). Plants were grown in a grid with equal distances between the positions of 12 rows × 60 columns. This grid was divided into three blocks of 12 ×20 each, which contained a single replicate of each RIL (1 × 100 lines) and four replicates for each CSL (4 × 32 lines) in a randomised complete block design. In a second separate experiment four replicates of each of the NILs (172 different genotypes in total; Supplementary Tables S7 and S8) segregating for chromosome 2 (37 genotypes with Col background and 39 with L*er* background) and chromosome 5 (45 genotypes with Col background and 51 with L*er* background) were grown in the same growth chamber under identical conditions. Here randomised complete blocks consisted of 12 ×30 positions that held two replicates of each genotype.

The number of days after planting at which the first flower opened was recorded (flowering time), at which time point the total length of the main inflorescence was measured (main stem length). Flowering time was corrected for germination date based on daily taken RGB-images by an automated camera system. The day at which the first green leaf could be detected was considered day zero. After three months, the experiment was terminated and plants not flowering by that time were considered outliers due to technical causes and removed from data analysis. Further outliers were determined by image analysis of individual plant growth performance and monitoring reports made during the experiment. Eventually, for most CSL genotypes at least ten replicates were analysed, with a few exceptions of which the CSL consisting of Chr1^L*er*^/Chr2^L*er*^/Chr3^Col^/Chr4^L*er*^/Chr5^Col^ was most extreme with only four replicates (Supplementary Table S9). For the NILs and RILs only genotypes for which at least two plants were available for each phenotype were included for data analyses (Supplementary Tables S9 and S10).

### Statistical analyses

The phenotypic data of the RILs and the NILs was corrected for environmental effects using the R package SpATS (Rodríguez-Álvarez et al. 2018). The script was adapted to our experimental setup, where population and block were included as fixed terms in the model while genotype, row and column were in the random part of the model. The geno.decomp option of SpATS was used to allow for heterogeneous genetic variances for the different populations (respectively the CSLs and RILs in the first experiment and the four different NIL panels in the second experiment). With this model the best linear unbiased predictions (BLUPs) were obtained for the NILs and the RILs (Supplementary Tables S11 and S12).

The BLUPs of the NILs and RILs were used as input for the QTL analyses with linear mixed models in Genstat 18th edition. The 676 single feature polymorphism (SFP) markers for the RILs were obtained from previously published data (Singer et al. 2006). Markers with a physical distance of roughly 1 Mbp, corresponding to approximately 5 cM genetic distance in Arabidopsis, were selected (Huang et al. 2011). Genetic predictors between markers were calculated by interval mapping with a step size of 5 cM to bridge any large gaps. For the QTL analyses default settings were used, with minimum cofactor proximity of 50 cM, minimum separation for selected QTLs of 30 cM and Li and Ji threshold settings with genome wide significance levels of α = 0.05 (Li and Ji 2005). Initially a single QTL model was fitted. QTLs of the initial analyses were included in the model as cofactors to test for additional QTLs. The QTLs detected and the –log10(p-values) of this composite interval mapping method are reported (Supplementary Tables S13 and S14). The support intervals were calculated as a drop of two units in the –log10(p-value) similar to a 2-LOD support interval.

The raw data of the CSLs was corrected for spatial trends with the SpATS R package, and the resulting spatial corrected raw data was used for further analyses (Supplementary Table S15). Individual trait values were preferred over BLUPs for the analyses of the CSLs to increase the degrees of freedom. Either all (for the analyses of the complete CSL set) or a subset (all sCSLs or the sCSLs sharing a single recurrent parent) of the corrected raw data was analysed by applying a backward elimination approach in combination with a multiple linear regression model containing chromosome main effects and two- and three-way epistatic interactions (I).

1 $${y}_{{ir}}=\mu +\mathop{\sum }\limits_{k=1}^{5}{a}_{k}{x}_{{ik}}+\mathop{\sum }\limits_{k=1}^{5}\mathop{\sum }\limits_{l > k}^{5}{b}_{{kl}}{x}_{{ik}}{x}_{{il}}+\mathop{\sum }\limits_{k=1}^{5}\mathop{\sum }\limits_{l > k}^{5}\mathop{\sum }\limits_{m > l}^{5}{c}_{{klm}}{x}_{{ik}}{x}_{{il}}{x}_{{im}}+{\varepsilon }_{{ir}}$$

where ${y}_{{ir}}$ is the phenotype of genotype $i$ in replicate $r$, $\mu$ is the overall mean, ${a}_{k}$ is the additive effect for chromosome $k$, ${x}_{{ik}}$ is an indicator variable, with ${x}_{{ik}}=0{(x}_{{ik}}=1)$ if chromosome $k$ for genotype $i$ is L*er* (Col), ${b}_{{kl}}$ are the effects for the two-way interactions between chromosomes $k$ and $l$, ${c}_{{klm}}$ are the effects of the three-way interactions between chromosomes $k$, $l$, and $m$, and ${\varepsilon }_{{ir}}$ is the residual error for genotype $i$ in replicate $r$.

To test three-way epistatic effects, the multiple linear regression model including all main, two- and three-way interactions (I) was compared with a model including main and two-way interactions (II) with backward selection of the AIC criterion using the stepAIC function of the MASS package (with α = 5.10^−5^ to correct for multiple testing) (Venables; and Ripley 2002).

2 $${h}_{0}:\,{y}_{{ir}}=\mu +\mathop{\sum }\limits_{k=1}^{5}{a}_{k}{x}_{{ik}}+\mathop{\sum }\limits_{k=1}^{5}\mathop{\sum }\limits_{l > k}^{5}{b}_{{kl}}{x}_{{ik}}{x}_{{il}}+{\varepsilon }_{{ir}}$$

A second step of parameter reduction was used to select the significant two-way interactions for the model with a similar significance threshold. Here, a model resulting from backward selection (IV) was compared to a model including only main effects (III):

3 $${h}_{0}:\,{y}_{{ir}}=\mu +\mathop{\sum }\limits_{k=1}^{5}{a}_{k}{x}_{{ik}}+{\varepsilon }_{{ir}}$$

4 $${h}_{1}:\,{y}_{{ir}}=\mu +\mathop{\sum }\limits_{k=1}^{5}{a}_{k}{x}_{{ik}}+\mathop{\sum }\limits_{k=1}^{5}\mathop{\sum }\limits_{l > k}^{5}{b}_{{kl}}{x}_{{ik}}{x}_{{il}}+\sum _{(k,l,m)\in {S}_{3}}{c}_{{klm}}{x}_{{ik}}{x}_{{il}}{x}_{{im}}\,+{\varepsilon }_{{ir}}$$

Here ${S}_{3}$ represents the set of the earlier selected significant three-way interactions. Finally, the model including all significant two- and three-way interactions (VI) was tested versus a model consisting of only the mean and the residuals (V):

5 $${h}_{0}:\,{y}_{{ir}}=\mu +{\varepsilon }_{{ir}}$$

6 $${h}_{1}:\,{y}_{{ir}}=\mu +\mathop{\sum }\limits_{k=1}^{5}{a}_{k}{x}_{{ik}}+\sum _{(k,l)\in {S}_{2}}{c}_{{kl}}{x}_{{ik}}{x}_{{il}}+\sum _{(k,l,m)\in {S}_{3}}{c}_{{klm}}{x}_{{ik}}{x}_{{il}}{x}_{{im}}+{\varepsilon }_{{ir}}$$

Here, ${S}_{2}$ in ${h}_{1}$ represents the significant two-way interaction terms that were selected in the previous round. This backward selection eventually resulted in a model that included all significant three- and two-way interactions and main effects and all terms underlying the significant interaction terms independent of their own significance according to the principal of marginality.

A similar approach was used for the analyses of the sCSLs were only the main effects model (III) was compared with a model consisting of only the mean and the residuals (V).

For the detection of interactions with the recurrent parental background (either Col or L*er*) all sCSLs were subjected to a similar backward selection procedure. Model (II) was adapted for chromosome x background interactions (VII) and compared with a model for main effects only (V) to test for significant interaction effects between the chromosomes and the background.

7 $${h}_{1}:\,{y}_{{ir}}=\mu +\mathop{\sum }\limits_{k=1}^{5}{a}_{k}{x}_{{ik}}+b{z}_{i}+\,\mathop{\sum }\limits_{k=1}^{5}{c}_{k}{x}_{{ik}}{z}_{i}+{\varepsilon }_{{ir}}$$

Where $b$ is the estimated background effect, ${z}_{i}$ is an indicator variable, with ${z}_{i}=0$ ${(z}_{i}=1)$ if the background $i$ is L*er* (Col), ${c}_{k}$ are the effects for the interaction between chromosome $k$ and the genetic background. Here significance thresholds were set to α = 1.10^−3^ to correct for multiple testing.

## Results

Here, we report on the construction and application of a complete set of CSLs resulting from a cross between the Arabidopsis accessions Columbia-0 (Col-0) and Landsberg *erecta* (L*er*) (Fig. 1 and Supplementary Fig. S1). Two of the 32 CSLs resemble the identical genotype of the original parents, albeit both in the cytoplasmic background of the inducer line now (*viz*. Col-0). However, ten (2 × 5, reciprocally) CSLs contain a single substituted chromosome (sCSL), whereas the other twenty (2 × 10, reciprocally) CSLs contain multiple substituted chromosomes. To demonstrate the enhanced power of complete CSL panels in genetic mapping and epistatic analyses, the complete panel was grown in a climate-controlled growth chamber under short day conditions. In order to compare the performance of CSL mapping with conventional linkage analysis a population of RILs derived from the same accessions was grown simultaneously (Lister and Dean 1993). To allow a fair comparison between population types an equal number of plants (i.e. #lines × #replicates) were analysed while warranting detection power of major and moderate-effect QTLs in the RIL population (Keurentjes et al. 2007). The marker density of the RIL population was sufficient to detect all crossovers and construct a high-resolution map (<1 cM). All plants were phenotyped for flowering time (days after germination) and main stem length (mm) at the moment of opening of the first flower.

### Detection of additive and epistatic effects in single-chromosome substitution lines

**Fig. 2:**
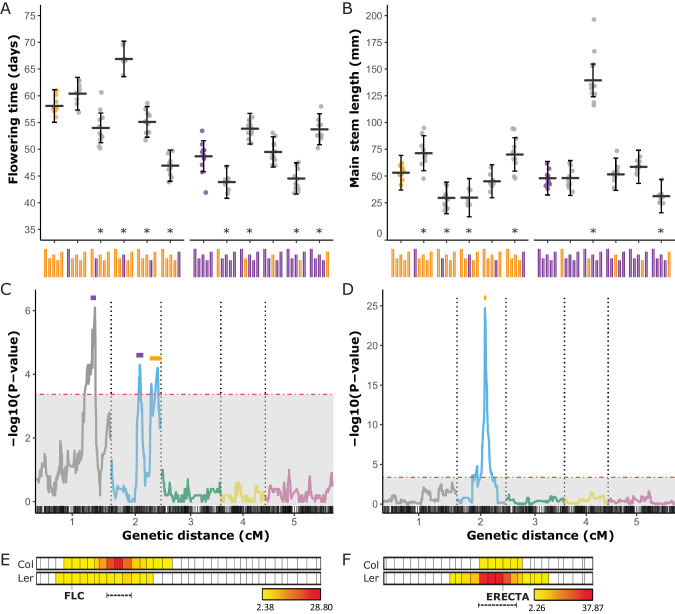
Mapping and validation of single-chromosome substitution effects. Flowering time (**A**) and main stem length (**B**) of sCSLs and their recurrent parents. Each dot represents the spatial corrected trait value of an individual of the genotype indicated below the x-axis. Horizontal bars indicate BLUPs with 95% confidence intervals shown as vertical bars (Supplementary Table S15). Asterisks denote significant effects. QTL plots for flowering time (**C**) and main stem length (**D**) as mapped in a RIL population. –log10(P) values for each chromosome are displayed in different colours, while the horizontal red dashed line represents the significance threshold. Support intervals for the QTLs are indicated by coloured bars according to effect sign (orange: +^Col-0^, and purple: +^L*er*^). The x-axis indicates chromosome numbers below a rug profile of the marker positions in cM distance. Heatmap plots of the effect strength of reciprocal chromosome five introgression NILs on flowering time (**E**) and chromosome two introgression NILs on main stem length (**F**). In both panels the upper row represents NIL mapping in a Col background, whereas the lower row represents NIL mapping in a *Ler* background. Vertical lines indicate marker positions in cM. Colour intensity from yellow to red specifies the strength of significant effects. Dashed lines with brackets below the heatmap indicate support intervals. FLC and ERECTA tick marks indicate the position of obvious candidate genes explaining variation in flowering time and main stem length, respectively.

In accordance with the use of conventional consomic strains the additive effect of a single substituted chromosome in comparison to the non-substituted recurrent parental genotype can be analysed. Moreover, since we have generated sCSLs in both recurrent parental backgrounds we can also specifically assess the contribution of epistatic effects to phenotypic variation (Chandler et al. 2013). Using a regression model obtained via a backward elimination procedure, significant effects on flowering time were detected for the substitution of the L*er* chromosomes 2, 3, 4 and 5 in the Col background (Fig. 2A; Table 1; Supplementary Table S1). Similarly, significant effects on main stem length were observed for the substitution of the L*er* chromosomes 1, 2, 3 and 5 in the Col background (Fig. 2B; Table 1; Supplementary Table S1). However, in contrast to the reciprocal exchange, the substitution of Col chromosome 3 in a L*er* background displayed no significant effect on flowering time, while the substitution of chromosome 1 did. Likewise, the substitution of the Col chromosomes 1 and 3 in a L*er* background had no significant effect on main stem length, while substitution of these chromosomes in a Col background led to significant differences. In addition to these qualitative background differences, the quantitative effect sizes of the reciprocal substitutions differed substantially. Although the largest effect on main stem length was caused by a substitution of chromosome 2 in both backgrounds, the size of the effect differed approximately four-fold. Furthermore, flowering time was mainly affected by substitution of chromosome 5 in the Col-0 background, whereas the largest effect in the L*er* background was obtained by the substitution of chromosome 2. These differences indicate both qualitative as well as quantitative interaction effects of single chromosome substitutions with the remainder of the genome. Indeed, when the substituted chromosomes and the recurrent background were both included in the regression model, significant interactions of most chromosomes with their background were detected for both traits (Fig. 2A, B; Table 1).

**Table 1:** Regression models for different CSL populations explaining variation in flowering time and main stem length.

Population	Background	Flowering time	Main stem length
5 sCSLs + Col parent	Col	*Chr2* *+* *Chr3* *+* *Chr4* *+* *Chr5*	*Chr1* *+* *Chr2* *+* *Chr3* *+* *Chr5*
5 sCSLs + L*er* parent	L*er*	*Chr1* *+* *Chr2* *+* *Chr4* *+* *Chr5*	*Chr2* *+* *Chr5*
10 sCSLs + both parents	Col + L*er*	*Chr1* *+* *Chr2* *+* *Chr3* *+* *Chr4* *+* *Chr5* + *BG* *+* *Chr3:BG* *+* *Chr4:BG* *+* *Chr5:BG*	*Chr1* *+* *Chr2* *+* *Chr3* *+* *Chr5* + *BG* *+* *Chr2:BG* *+* *Chr3:BG*
32 CSLs	Col + L*er*	*Chr1* *+* *Chr2* *+* *Chr3* *+* *Chr5* *+* *Chr1:Chr3* *+* *Chr1:Chr5* *+* *Chr3:Chr5*	*Chr1* *+* *Chr2* *+* *Chr3* *+* *Chr5* *+* *Chr1:Chr2* *+* *Chr1:Chr5* *+* *Chr2:Chr5* *+* *Chr3:Chr5* *+* *Chr1:Chr2:Chr5*

Populations consist of CSLs with only a single substituted chromosome in a particular background plus their recurrent parent, a set of all sCSLs plus recurrent parents, or the complete set of CSLs, including parental genotypes. Regression models contain only backward selected parameters significantly contributing to explained variance. The parameters *Chr1*, *Chr2*, *Chr3*, *Chr4* and *Chr5* denote additive effects of individual chromosomes whereas *BG* denotes background effects. Parameter components separated by a colon indicate interaction effects.

### Chromosome substitution lines offer improved mapping power

Strikingly, the number of QTLs detected in the RIL population using conventional composite interval mapping (CIM) was much lower than in the sCSL panel (Supplementary Table S1), as was also previously observed for rodents (Buchner and Nadeau 2015). For flowering time two significant QTLs were detected on chromosome 2 and an additional one on chromosome 1 but no QTLs were detected on any of the other three chromosomes, consistent with previous studies (Ungerer et al. 2003) (Fig. 2C). Furthermore, variation in main stem length in the RIL population is largely explained by a single QTL on chromosome 2, most likely reflecting allelic variation of the *ERECTA* locus (Ungerer et al. 2002) (Fig. 2D). So, in concordance with studies of sCSLs in rodents, our CSL population outperformed traditional linkage mapping in RILs in QTL detection power for all traits analysed.

### Validation and finemapping of CSL-QTLs with Near Isogenic Lines

Despite the high detection power, CSLs inherently offer a low resolution since QTLs can only be mapped to entire chromosomes due to the lack of recombination. A consequence of this is that intrachromosomal epistasis cannot be detected in CSLs unless they are allowed to recombine in subsequent crosses. To overcome this drawback a reciprocal genome-wide coverage set of near-isogenic lines (NILs) was generated. These were produced by backcrossing sCSLs to one of the recurrent parental accessions and subsequent DH production of recombinant F_1_ gametes, as described for the generation of CSLs. In total 413 NILs with either a single or multiple introgressions were generated of which 219 contained a L*er* introgression in a Col background and 194 contained a Col introgression in a L*er* background, as determined by marker-assisted genotyping (Supplementary Table S3). This genetic resource serves to validate and fine-map detected QTLs in the CSLs and confirm possible epistatic interactions with the genetic background.

To demonstrate the complementing value of this NIL population, a subset of reciprocal NILs covering the chromosomes 2 (76 NILs) and 5 (96 NILs) were grown in similar conditions as the CSLs and RILs. The substitution of chromosome 2 had the largest effect on main stem length, with two-fold longer stems in genotypes carrying a Col chromosome 2 (Fig. 2F). Fine-mapping of this chromosome in the reciprocal NILs resulted in a support interval of 9.1–16.5 Mbp for the Col set, while this was much narrower in the L*er* set, 9.9–11.3 Mbp. This coincides well with the support interval of the QTL mapped in the RIL population (11.1–11.7 Mbp, Fig. 2D) and covers the position of the obvious candidate gene *ERECTA* at 11.2 Mbp. A similar resolution, support interval 7.3–8.8 Mbp and 8.0–9.7 Mbp for Col and L*er* NILs respectively, could be obtained for the fine-mapping of the chromosome 5 QTL for flowering time. Although the strong *FLOWERING LOCUS C* (*FLC*) was located on the same chromosome arm this gene is positioned outside the QTL support interval and can, therefore, not be considered as a candidate gene (Fig. 2E). Surprisingly, despite a ten-day delay in flowering time in sCSLs in which a L*er* chromosome 5 is substituted in a Col background, this QTL was not detected in the RILs (Fig. 2C). This might be explained by the confounding effects of genetic interactions in the analysis of main effects in the RIL population. Alternatively, the haploidization process that CSLs and NILs underwent might have led to chromosomal rearrangements but full-genome sequence analysis of all CSLs did not indicate any rearrangements or other anomalies.

### Epistasis explains a large part of the genetic variation in quantitative traits

An interesting observation from the analysis of the reciprocal NIL sets is the difference in mapping power. The effect on flowering time of a L*er* chromosome 5 substitution in a Col background (∆FT = −7.4 days) is much larger than vice versa (∆FT = + 4.5 days). Likewise, the effect on main stem length of a Col chromosome 2 substitution in a L*er* background is almost eightfold larger than vice versa. These differences might reflect discrepancies in effect sizes relative to the recurrent parent’s trait value, which might be the result of an accumulation of additive effects, or could indicate a dependency on epistatic interactions. Although the limited set of reciprocal sCSLs also indicates the presence of epistasis, both chromosome 2 and 5 were identified to interact with the background in determining main stem length and flowering time, respectively, the specific origin of these genetic interactions can only be identified by comparing CSLs with multiple substituted chromosomes.

The importance of genetic interactions, relative to the additive effects of single loci, on the phenotypic expression of a trait is part of a long lasting debate (Carlborg and Haley 2004; Fisher 1919; Nelson et al. 2013) and multiple studies have reported on models including epistasis that explain more variation (Bloom et al. 2015) and have a better predictive power (Forsberg et al. 2017) compared to models including only main effects. However, the unbiased testing of epistasis as a source of natural variation is statistically challenging since increasing levels of interaction decrease the number of observations for each genotypic class, which drains the power to detect interacting loci. Furthermore, in most standard mapping populations undetected QTLs are added to the error term. Finally, overfitting of a model can become a problem due to the close to an infinite number of allelic combinations in a segregating recombinant biparental population. Therefore, most statistical models only include main additive effects and the interactions between them, leaving part of the heritable variation unexplained (Carlborg and Haley 2004). Completely balanced CSL panels, however, offer the unique opportunity to analyse the relatively limited number of all possible genotypic combinations in a full factorial design and as such provide a more realistic view on the complexity of quantitative trait regulation.

**Fig. 3:**
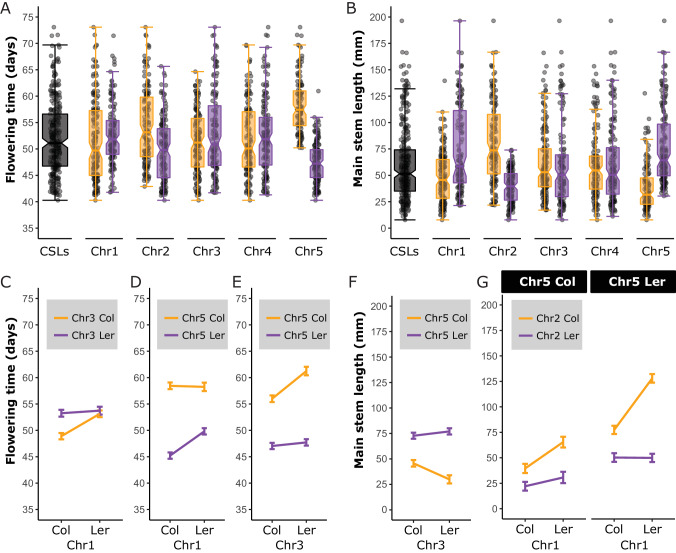
Detection of interchromosomal interaction effects in a complete CSL panel. Notched box-and-whisker plots of the complete panel of CSLs for flowering time (**A**) and main stem length (**B**). Each dot represents the spatial corrected trait value of an individual plotted in relation to all other individuals (grey boxes) or categorised according to its genotype for the chromosome indicated on the X-axis (orange boxes: Col; purple boxes: L*er*). **C**–**G** Regression predicted effect plots of epistatic interactions identified with backward selection models. **C**–**E** Two-way interactions explaining variation in flowering time. **F** Two-way interaction explaining variation in main stem length. **G** Three-way interaction explaining variation in main stem length. Error bars represent the 95% confidence intervals of the predicted effect.

Since clear indications of genetic interactions between chromosomes were obtained from the analysis of reciprocal CSLs and NILs, a regression analysis using a backward elimination strategy on data of the complete CSL panel (Fig. 3A, B) was performed to quantify the contribution of epistasis to the phenotype. Using a similar regression approach as was used to test the sCSLs for background interactions, significant chromosome interactions were included in the final model. For flowering time, significant two-way interactions were detected between chromosome 1 and 3, 1 and 5, and 3 and 5 (Fig. 3C–E), which partly explain the major effect of genotypic variation of chromosome 5 (Fig. 3A). For main stem length a significant three-way interaction between the chromosomes 1, 2 and 5 was detected, while a significant two-way interaction was detected between chromosome 3 and 5 (Fig. 3F, G).

Although in general main effect sizes are considered to be larger than interaction effects, here the interaction effect of three chromosomes on main stem length is of similar size as the most effective substitution of a single chromosome (Fig. 2B). Most notable for this three-way interaction is a more than 65% increase in main stem length of one genotypic class (Chr1^Ler^/Chr2^Col^/Chr5^Ler^) over any of the other seven genotypic classes (Fig. 3G). The importance of epistasis is also demonstrated by a comparison of regression models which either include or exclude epistatic interactions. An inclusive model displays a superior predictive power (R^2^ = 0.835) over a model in which epistatic interactions are not considered (R^2^ = 0.760; Supplementary Fig. S2). Finally, the impact that genetic interactions can have on the phenotype is illustrated by a case of antagonistic epistasis between chromosome 3 and 5, where the substitution of a Col chromosome 3 with that of L*er* resulted in opposite effects on main stem length, depending on the genotype of chromosome 5.

## Discussion

Our results show that a relatively large part of the observed variation in the analysed quantitative traits can be explained by epistatic interactions. The power to detect these interactions and estimate their effect sizes is greatly enhanced by analysing a complete panel of CSLs, which also includes lines in which multiple chromosomes are substituted. The notion that even for traits dominated by major effect loci (e.g. *ERECTA* in main stem length) epistatic interactions can be revealed, and given the small size of this population, CSL mapping holds great promises for many other quantitative traits in Arabidopsis. There is no reason to assume that similar results cannot be obtained in other species, although larger genome sizes (i.e. higher chromosome numbers) might require the simultaneous substitution of two or more chromosomes. For instance, a complete CSL panel of cucumber (*n* = 7), brassica (*n* = 9), maize (*n* = 10) or tomato or rice (*n* = 12) would require 128, 512, 1024 and 4096 unique lines, respectively. Moreover, polyploid crops such as wheat (2*n* = 6× = 42) would make the construction of complete CSL panels progressively more complex. However, partial libraries in which chromosome genotype combinations are evenly represented in smaller subsets may be sufficient to detect main effects and simple two-way interactions. Furthermore, the homologous Brassicae *DMC1* gene was used for RNA interference, suggesting that meiotic suppression may be more universally applicable. Alternatively, other genes essential during meiotic division can be targeted or chemical suppression may reduce the crossover frequency (Sanchez-Moran et al. 2004). Similarly, a modified CenH3 also induced haploidization in maize (Meng et al. 2022) although other methods may apply for other species (Jacquier et al. 2020). Hence, the development of CSL panels for other species than Arabidopsis is a realistic alternative for conventional mapping populations and may greatly enhance our understanding of the contribution of epistasis to natural variation in quantitative traits.

### Supplementary information

Supplemental material

Dataset 1

## Data Availability

All data is available from the supplementary materials and deposited at the DRYAD repository (10.5061/dryad.2bvq83c08). Materials will be donated to the appropriate stock centres.
