# Supplementary material for: A complete chromosome substitution mapping panel reveals genome-wide epistasis in Arabidopsis
Source: Heredity (Edinb). 2024 Jul 9;133(3):198–205. doi: 10.1038/s41437-024-00705-1 (PMC11350127; doi:10.1038/s41437-024-00705-1)
Supplement: Supplementary file 1 — Supplemental material [file 41437_2024_705_MOESM1_ESM.docx]

**Title:**

A complete chromosome substitution mapping panel reveals genome-wide epistasis in Arabidopsis

**Authors:**

Cris L. Wijnen^1†^, Ramon Botet^1†^, José van de Belt^1^, Laurens Deurhof^1^, Hans de Jong^1^, C. Bastiaan de Snoo^2^, Rob Dirks^2,3^, Martin P. Boer^4^, Fred A. van Eeuwijk^4^, Erik Wijnker^1§^, Joost J.B. Keurentjes^1§*^

^*^ Correspondence to:

J.J.B. Keurentjes,

Droevendaalsesteeg 1,

6708 PB Wageningen,

The Netherlands,

+31317483149

[joost.keurentjes@wur.nl](mailto:joost.keurentjes@wur.nl)

**This PDF file includes:**

Figures S1 to S2

Tables S1 to S2


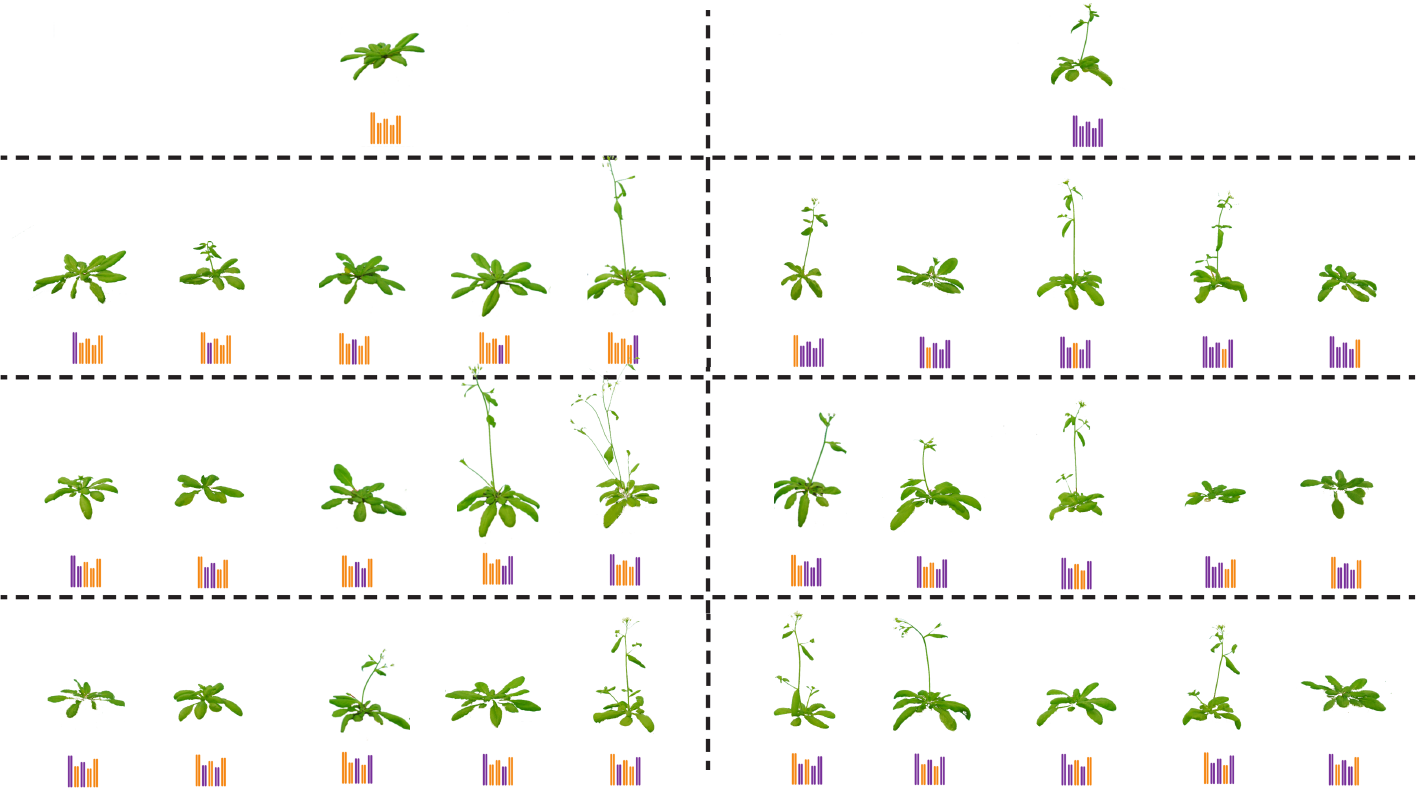


**Figure S1: Photographic presentation of phenotypic variation in a complete panel of CSLs.** Each image depicts a representative phenotype of the genotype plotted below it. Arabidopsis genomes of each of the 32 CSLs are represented by five homozygous chromosomes derived from either the Col-0 (orange) or L*er* (purple) accession. Depicted plants are of identical age and images were taken at 23 days after sowing in long day (16h light) conditions.


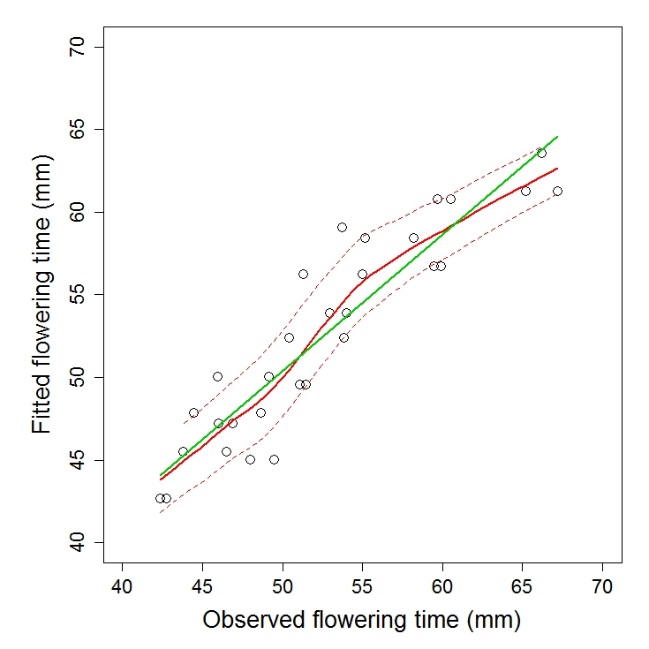

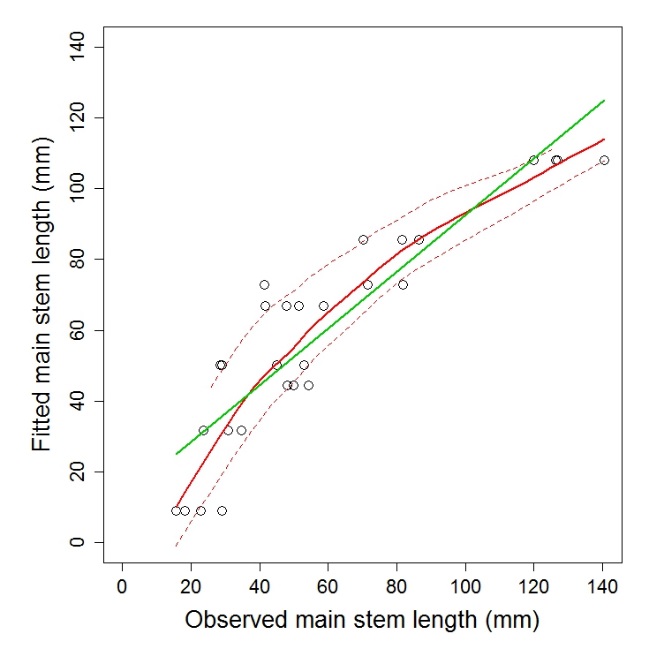

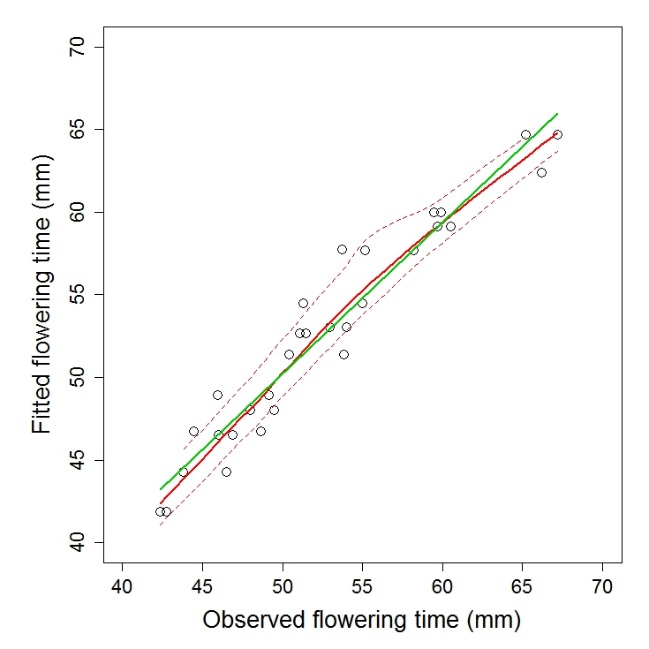

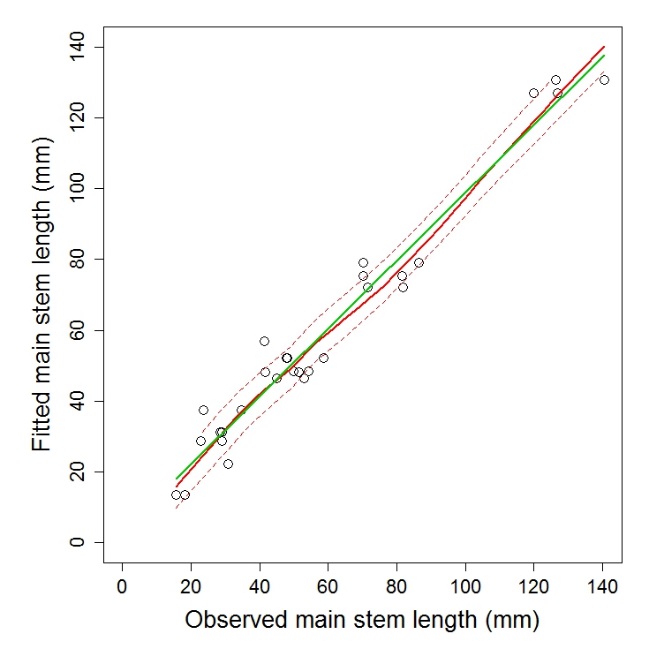


D

B

A

C

**Figure S2: Scatterplots of predicted trait values for models without and with interaction terms.** The x-axis shows the observed mean phenotypic values of the CSls, and the y-axis the predicted values according to the corresponding model. A-B) Prediction of models without interaction terms for flowering time (FT ~ Chr1 + Chr2 + Chr3 + Chr5) and main stem length (MSL ~ Chr1 + Chr2 + Chr5), respectively. C-D) Prediction of the epistatic models for flowering time (FT ~ Chr1 + Chr2 + Chr3 + Chr5 + Chr1:Chr3 + Chr1:Chr5 + Chr3:Chr5) and main stem length (MSL ~ Chr1 + Chr2 + Chr3 + Chr5 + Chr1:Chr2 + Chr1:Chr5 + Chr2:Chr5 + Chr3:Chr5 + Chr1:Chr2:Chr5), respectively. For each plot, the linear regression is shown in green, while the red line represents a trend line of the data including a LOESS-confidence interval between the dashed lines.

**Table S1: Detailed overview of main and interaction effects detected in CSL populations.** For each significantly detected effect the trait for and population type in which it was detected is given. FT, flowering time; MSL, main stem length; Population type, sCSL: five sCSLs plus their indicated recurrent parent, sCSLs: all 10 sCSLs plus their recurrent parents, All CSLs: all 32 CSLs including the recurrent parental genotypes; Background genotype, the recurrent genotype for the sCSL populations; Chromosome number, the chromosomes for which main or interaction effects were detected, BG: effect of recurrent genotype in sCSL comparisons; Effect size, main effects: effect of background genotype or the substitution of a L*er* chromosome with a Col chromosome (∆Col-L*er*), interaction effects: the average effect of the substitution of either one of the interacting chromosomes or background compared to the population mean, FT (days), MSL (mm); s.e., standard error of the effect size in the same units; Explained variance, proportion of the population variance explained by each effect; Significance, the significance of the effect.

| **Trait** | **Population**  **type** | | **Background**  **genotype** | | **Chromosome**  **number** | | **Effect size** | | **s.e.** | **Explained variance (%)** | | **Significance**  **(P-value)** |  |
| --- | --- | --- | --- | --- | --- | --- | --- | --- | --- | --- | --- | --- | --- |
| FT | | sCSL | | Col | | II | 5.4 | 0.9 | | 2.33 | 8.7E-08 | | |
|  | |  | |  | | III | -7.8 | 1.1 | | 35.39 | 1.9E-09 | | |
|  | |  | |  | | IV | 4.3 | 0.9 | | 0.05 | 9.7E-06 | | |
|  | |  | |  | | V | 12.7 | 0.9 | | 49.79 | < 2E-16 | | |
|  | |  | |  | |  |  |  | |  |  | | |
|  | | sCSL | | L*er* | | I | -5.5 | 0.8 | | 23.99 | 1.3E-08 | | |
|  | |  | |  | | II | 4.8 | 0.8 | | 18.19 | 3.1E-08 | | |
|  | |  | |  | | IV | -4.6 | 0.8 | | 24.90 | 6.7E-08 | | |
|  | |  | |  | | V | 4.7 | 0.8 | | 11.77 | 2.3E-07 | | |
|  | |  | |  | |  |  |  | |  |  | | |
|  | | sCSLs | |  | | I | -3.6 | 0.7 | | 1.83 | 1.3E-06 | | |
|  | |  | |  | | II | 4.7 | 0.7 | | 27.66 | 2.8E-10 | | |
|  | |  | |  | | III | -9.2 | 1.1 | | 0.41 | 5.5E-14 | | |
|  | |  | |  | | IV | 2.8 | 0.9 | | 0.80 | 2.2E-03 | | |
|  | |  | |  | | V | 11.2 | 0.9 | | 41.02 | < 2E-16 | | |
|  | |  | |  | | BG | 6.1 | 2.1 | | 0.30 | 5.0E-03 | | |
|  | |  | |  | | III:BG | 10.3 | 1.2 | | 11.55 | 3.2E-13 | | |
|  | |  | |  | | IV:BG | 6.8 | 1.1 | | 2.51 | 7.0E-09 | | |
|  | |  | |  | | V:BG | 5.9 | 1.1 | | 2.72 | 6.8E-07 | | |
|  | |  | |  | |  |  |  | |  |  | | |
|  | | All CSLs | |  | | I | -1.5 | 0.6 | | 0.76 | 9.5E-03 | | |
|  | |  | |  | | II | 4.7 | 0.3 | | 10.81 | < 2E-16 | | |
|  | |  | |  | | III | -7.0 | 0.6 | | 1.59 | < 2E-16 | | |
|  | |  | |  | | V | 11.2 | 0.5 | | 62.80 | < 2E-16 | | |
|  | |  | |  | | I:V | 3.8 | 0.7 | | 1.85 | 5.8E-08 | | |
|  | |  | |  | | I:III | 4.8 | 0.7 | | 3.25 | 1.2E-11 | | |
|  | |  | |  | | III:V | 4.6 | 0.7 | | 2.50 | 1.1E-10 | | |
|  | |  | |  | |  |  |  | |  |  | | |
| MSL | | sCSL | | Col | | I | -23.0 | 4.1 | | 21.61 | 8.9E-07 | | |
|  | |  | |  | | II | 19.4 | 4.0 | | 19.55 | 1.1E-05 | | |
|  | |  | |  | | III | 19.5 | 5.0 | | 16.55 | 2.6E-04 | | |
|  | |  | |  | | V | -23.0 | 4.0 | | 16.24 | 4.5E-07 | | |
|  | |  | |  | |  |  |  | |  |  | | |
|  | | sCSL | | L*er* | | II | 88.7 | 4.0 | | 86.48 | < 2E-16 | | |
|  | |  | |  | | V | -21.0 | 4.3 | | 3.68 | 8.4E-06 | | |
|  | |  | |  | |  |  |  | |  |  | | |
|  | | sCSLs | |  | | I | -14.5 | 3.3 | | 10.67 | 2.5E-05 | | |
|  | |  | |  | | II | 21.6 | 4.3 | | 34.27 | 1.6E-06 | | |
|  | |  | |  | | III | 21.7 | 5.4 | | 3.32 | 1.0E-04 | | |
|  | |  | |  | | V | -22.6 | 3.2 | | 18.15 | 1.1E-10 | | |
|  | |  | |  | | BG | -48.8 | 7.5 | | 1.88 | 2.2E-09 | | |
|  | |  | |  | | II:BG | 63.5 | 5.6 | | 16.88 | < 2E-16 | | |
|  | |  | |  | | III:BG | 25.5 | 6.5 | | 1.73 | 1.5E-04 | | |
|  | |  | |  | |  |  |  | |  |  | | |
|  | | All CSLs | |  | | I | -25.8 | 3.5 | | 13.18 | 2.5E-12 | | |
|  | |  | |  | | II | 17.5 | 3.2 | | 33.85 | 7.3E-08 | | |
|  | |  | |  | | III | 15.2 | 2.6 | | 0.00 | 6.7E-09 | | |
|  | |  | |  | | V | -29.1 | 3.5 | | 24.06 | 1.6E-15 | | |
|  | |  | |  | | I:II | 17.2 | 5.0 | | 8.06 | 7.1E-04 | | |
|  | |  | |  | | I:V | 24.8 | 4.6 | | 0.24 | 1.7E-07 | | |
|  | |  | |  | | II:V | 9.6 | 4.4 | | 2.76 | 2.8E-02 | | |
|  | |  | |  | | III:V | 19.3 | 3.3 | | 1.55 | 1.4E-08 | | |
|  | |  | |  | | I:II:V | 33.8 | 6.6 | | 1.35 | 4.8E-07 | | |

**Table S2: Detailed overview of the QTLs detected in RIL and NIL populations.** For each significantly detected QTL the trait for and population type in which it was detected is given. FT, flowering time; MSL, main stem length; Background genotype, for the NILs the recurrent background is given, equal allele frequencies are assumed for RILs; Chromosome number, the chromosome on which the QTL was detected; Position, position on the chromosome where the strongest association was detected; Support interval, support intervals were calculated as a drop of two units in the –log10(p-value) surrounding the position of the most significant association; Effect size, effect of the homozygous substitution of a L*er* genotype with a Col genotype at the QTL (∆Col-L*er*), FT (days), MSL (mm); s.e., standard error of the effect size in the same units; Explained variance, proportion of the total population variance explained by each QTL; Significance, the significance of the strongest association detected.

| **Trait** | **Population**  **type** | | **Background**  **genotype** | | **Chromosome**  **number** | **Position**  **(Mbp)** | **Support**  **interval (Mbp)** | **Effect**  **size** | | **s.e.** | **Explained**  **variance (%)** | **Significance**  **(-log10(p))** |
| --- | --- | --- | --- | --- | --- | --- | --- | --- | --- | --- | --- | --- |
| FT | | RILs | | N.A. | I | 23.8 | 22.2 - 24.1 | -2.14 | 0.64 | | 8.4 | 6.1 |
|  | |  | |  | II | 11.2 | 10.1 - 12.4 | -2.68 | 0.67 | | 13.3 | 4.3 |
|  | |  | |  | II | 18.3 | 15.3 - 19.5 | 4.18 | 0.63 | | 32.4 | 4.2 |
|  | | NILs | | Col | V | 8.0 | 7.3 - 8.8 | 7.38 | 0.65 | | 78.2 | 28.8 |
|  | | NILs | | L*er* | V | 8.8 | 8.0 - 9.7 | 4.47 | 0.88 | | 39.7 | 6.4 |
|  | |  | |  |  |  |  |  |  | |  |  |
| MSL | | RILs | | N.A. | II | 11.2 | 11.1 - 11.7 | 34.95 | 3.05 | | 64.0 | 24.7 |
|  | | NILs | | Col | II | 11.3 | 9.1 - 16.5 | 8.53 | 2.30 | | 33.9 | 3.7 |
|  | | NILs | | L*er* | II | 10.6 | 9.9 - 11.3 | 65.17 | 5.02 | | 85.5 | 37.9 |
